# Actionable partnerships for shaping healthcare’s future by closing the equitable access gap and delivering meaningful change

**DOI:** 10.1057/s41271-025-00578-6

**Published:** 2025-07-01

**Authors:** Manoj Monga, Denise Asafu-Adjei, Sirikan Rojanasarot, Emmanuel Ezekekwu, Natalie Edwards, Kathryn Unger, Erin Turner, Samir Bhattacharyya

**Affiliations:** 1 University of California, San Diego, San Diego, United States; 2 University of Chicago Medicine, Chicago, United States; 3 Boston Scientific, Marlborough, United States; 4 Health Services Consulting Corporation, Boxborough, MA, United States

**Keywords:** Health equity, Corporate social responsibility, Disparities, Diversity

## Abstract

Putting health equity into practice requires deliberate, longitudinal work by numerous stakeholders and a multi-sectoral strategy that simultaneously engages academic, governmental, and corporate entities. Corporate social responsibility in healthcare research and product development is a critical step to ensure that everyone benefits from diagnostics and treatments, and that all patients’ needs are met. Herein we describe activities ongoing at a global healthcare technology company in order to help close the treatment gap and improve equitable access to care. Published literature was used to inform a framework for organizing the activities into four initiatives: (1) raising awareness of the implications of the status quo; (2) implementing equitable patient-centered innovation; (3) building a more diverse workforce; and (4) generating the evidence base for equitable care. The four ongoing initiatives illustrate specific efforts that healthcare professionals and corporations can make to help close the health equity gap and shape the future of healthcare.

## Key messages

- Incorporating health equity practices requires a joint effort by multiple stakeholders, including corporate social responsibility (CSR) in medical research and development
- Four initiatives for closing the health equity gap illustrate what is possible through intentional partnership: (1) raising awareness of the implications of the status quo; (2) implementing equitable patient-centered innovation; (3) building a more diverse workforce; and (4) generating the evidence base for equitable care.
- Using diversity planning, health equity, and CSR strategies, businesses are strongly positioned to further the movement toward health equity.

## Introduction

The mandate to put health equity into practice has intensified for all healthcare organizations to contribute to the improvement of health and well-being in society at large, through diversity planning and corporate social responsibility (CSR) strategies and other approaches to achieve equitable access to care and health equity [1]. The challenges of COVID-19 and latent social injustices, which have been prominently highlighted since 2020, underscored the urgency of working fervently to close the health equity gap [2]. Incorporating health equity practices is a joint effort by multiple stakeholders to tackle the deep societal roots of national and global historical disparities. By focusing on the needs of the underserved populations, we can find solutions with the collaborative efforts of the communities, businesses, academic institutions, and governments to positively influence health outcomes and move toward our health equity goals—equitable access to and utilization of high-quality healthcare and optimal health for all [3].

**Fig. 1:**
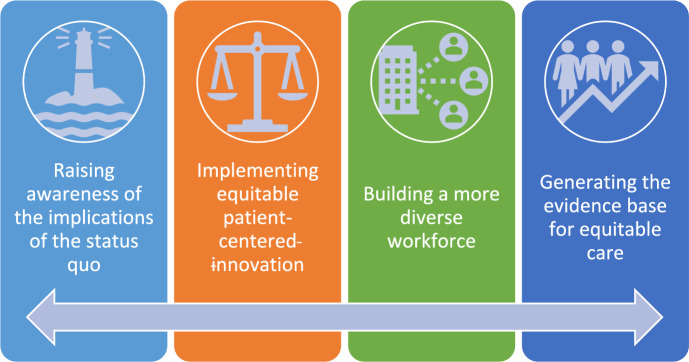
Four initiatives for delivering change

CSR in medical research and development is especially important as a critical step to ensure that everyone benefits from diagnostics and treatments, and that newly emerging technologies meet the needs across all populations. This is in contrast to continuing research and development that perpetuate historical structural biases and thus health disparities. Health equity activities empower physicians, healthcare providers, community stakeholders, healthcare researchers, and others to address healthcare disparities experienced by women, persons of color, Lesbian, Gay, Bisexual, Transgender, Queer, Intersex, and Asexual + all other identities (LGBTQIA +), and other underrepresented populations. Herein we describe activities ongoing at a global healthcare technology company (Boston Scientific), in collaboration with various healthcare stakeholders, particularly urologists, in order to help close the treatment gap, improve medical innovations, and equitable access to care. Published literature was used to inform a framework for organizing the activities into four initiatives: (1) raising awareness of the implications of the status quo; (2) implementing equitable patient-centered innovation; (3) building a more diverse workforce; and (4) generating the evidence base for equitable care (Fig. 1).

## Four initiatives for delivering change

### Initiative #1: Raising awareness of the implications of the status quo

The past five decades have seen great strides in terms of understanding the nature and scope of health disparities and their socioeconomic healthcare-related drivers in the United States (US) [4]. The Centers for Disease Control and Prevention’s (CDC’s) Behavioral Risk Factor Surveillance System study found that from 1993 to 2017, the Black-White gap showed significant improvement; however, health disparities are still a great concern [5]. The disproportionate burden of COVID-19 among Black and Hispanic populations in terms of prevalence, hospitalizations, and mortality exemplified these concerns [6–9]. Blacks, Hispanics, and other underrepresented populations had higher rates of underlying health conditions such as diabetes, hypertension, obesity, and cardiovascular disease that made them more vulnerable to COVID-19 [6–8]. COVID-19 deaths were 105% higher in Blacks, 15% higher in Hispanics, and 29% higher in other ethnic groups [9]. Due to systemic challenges related to the social determinants of health, the consequences of diabetes and obesity [10] and the risk of cancer [11] are also often much greater in underrepresented populations than in their non-Hispanic White patient counterparts. Substantial disparities in peripheral artery disease (PAD) treatment patterns between African-Americans and Hispanic-Americans compared to non-Hispanic White populations have been observed [12]. Blacks are amputated at twice the rate of Whites, and Hispanic-Americans are amputated at a rate 50% higher than their non-Hispanic White counterparts [12].

The mission of the R. Frank Jones Urological Society (RFJUS) is to support Black American urologists in their efforts to care for patients, as they exercise clinical excellence to achieve quality outcomes and reduce healthcare disparities for Black people with urologic conditions. In partnership with RFJUS and a local community organization, Boston Scientific sponsored a health equity initiative for prostate cancer screenings in New Orleans [13]. In addition, the company is collaborating with the Consortium on Disparities of Urologic Conditions (ConDUC) program, a non-profit organization whose mission is to develop strategies to eliminate health disparities and improve outcomes in populations with urologic diseases through clinical trials and research.

Structural inequities underlying formal healthcare delivery must also be considered when aiming to deliver equitable healthcare for all. For example, one of the major issues contributing to the severe illness and death rate from COVID-19 in underrepresented groups was that these people often lived and worked in more high-risk environments and were on the frontlines during the pandemic [14]. The root causes of health inequity are diverse, complex, evolving, and interdependent; the underlying causes and conditions related to health inequity must be better understood and identified to inform interdisciplinary and effective interventions [15]. The lack of trust among underrepresented groups in medicine due to historical medical mistreatment and unethical medical experimentation is well-documented in the medical literature [16–22]. Decades of work implementing cultural competency training, patient-centered communication, community engagement, including more clinicians with similar backgrounds (discussed below), have improved trust [23].

Working more with health systems, hospitals, and colleagues locally and demonstrating transparency has also been proven to be more effective [24]. Social determinants of health such as accumulated conditions over time in the environments in which people are born, live, work and contribute to health inequities and influence health outcomes as well as quality of life [25]. Social interventions must incorporate these historical and present factors. The incorporation of the health equities into the Quintuple Aim to extend the Quadruple Aim also highlights the importance of integrating of higher quality care, a strong patient-centered experience, better community health outcomes, and an engaged clinician workforce that is not burned out [26].

Healthcare providers are very important and effective advocates for health equity change as they may command great respect and trust within their communities [27]. Providers from underrepresented communities and caring for those populations must be supported in their efforts on the front lines, as they are in excellent positions to identify local underlying factors, inspire improved health behaviors, and address healthcare inequity. In an effort to identify opportunities to support urologists, explore ways to improve diversity among urologists, and learn more about unmet community needs in striving for health equity, Boston Scientific partnered with the extended clinician community at a 2-day inaugural “Urology and Social Responsibility” meeting at the University of California, San Diego (UCSD) [28]. Discussions regarding some of the challenges and opportunities that face society highlighted issues concerning addressing diversity, equity, and inclusion in urology, addressing gender equity in urology, and addressing wellness and burnout in urology. The event demonstrated how urologists can partner with businesses to focus societal attention on the burden of inequities in urological care.

### Initiative #2: Implementing equitable patient-centered innovation

Internal processes for innovation at healthcare technology companies must incorporate inclusive and equitable patient-centered technology. Ensuring patient centricity is at the forefront of any initiative that industry undertakes. Patient-centered technology means including patient-reported outcomes (PROs) and other humanistic parameters in clinical development along with expert healthcare providers and clinicians. In addition to clinical trial data generation, PROs need to be inclusive and equitable. The European Medicines Agency (EMA) is set to recommend the advancement of standards for clinical studies incorporating robust and meaningful patient experience data for regulatory submission. PROs are an important tool to demonstrate the inclusion of underrepresented populations in data collection reflective of diverse and multicultural populations to improve research and promote equitable healthcare for the benefit of all patients and the broader public. To ensure the successful and equitable adoption of health technology innovations, researchers must think beyond the device and the direct end user and must seek a more holistic understanding of broader patient needs and the intended context of use early in the design process and throughout the product lifecycle [29]. Equitable adoption of innovative technologies includes training for appropriate use, maintenance, and upgrades over time as next-generation technologies become available. The US Food and Drug Administration (FDA) proposed updating its Breakthrough Devices Program (BDP) in 2022 to broaden its eligibility to include devices that could promote and advance health equity. The company is thinking beyond the devices themselves by explicitly incorporating the needs of end users and broader stakeholders into its design framework. It actively includes diverse and underrepresented patient populations, multi-disciplinary clinical physicians, and specialists such as urologists in their product development process, understanding that these perspectives from various backgrounds result in improved innovative medical solutions and reflect the diverse populations they serve [30]. By coordinating with physicians grounded in their local communities that have often been left out of the innovation process, medical technology developers can ensure that the preferences and requirements for effective use of innovations by underrepresented populations are considered at the onset and throughout the innovation lifecycle. The creation of the Patient-Centered Outcomes Research Institute (PCORI) is another example of the value of incorporating the patient’s perspective. PCORI is widely acknowledged as a leader in driving US clinical research to become more patient-centered and is “committed to ensuring that patients and other healthcare decision-makers have a seat at the table throughout the clinical research process — helping to prioritize research topics, design and conduct the studies and share the results” [31].

Inequities in access to new technologies are detrimental to public health [32]. Structural barriers can be mitigated with policy changes to more effectively address the wider social determinants of health, especially those related to access to innovations [33]. Advocacy through empowerment, education, and collaboration is essential for enabling more equitable utilization of and access to new diagnostics and treatments to reduce historical inequities [33].

Artificial intelligence (AI) will increasingly be applied within healthcare to help manage the complexity and burden of enormous amounts of data and aid in medical decision-making [34]. Key categories of AI applications already being employed by payers, health systems, and life sciences companies include diagnosis and treatment recommendations, patient engagement and adherence, and infrastructure activities [34]. AI can enable healthcare systems to achieve their ‘quadruple aim' by democratizing and standardizing a future of connected and AI augmented healthcare [35]. However, AI may provide opportunities and challenges for equity in healthcare [36]. Boston Scientific recognizes that appropriate deployment of AI technologies should work toward decreasing existing inequities [37]. While it holds tremendous promise, AI has the ability to perpetuate bias at scale and may compromise certain data privacy concerns in underrepresented groups [38]. The manner in which stakeholders in the innovation process operationalize equity recognition will be crucial to the viability and trustworthiness of algorithms in healthcare [38]. In its development of medical solutions and enhancing patient and physician experiences through AI real-time insights, the company is making efforts to consider equitable access and use of innovations, taking into account the various interconnected system-level relationships. A recent example is Boston Scientific’s BeatLogic™ machine learning-supported algorithm for wearable cardiac devices that securely monitors variations in heart rhythm across diverse patient populations, enabling fast physician notifications for earlier interventions and informing more equitable device designs [39]. Utilizing recognized CDC public health frameworks [40] for implementation of these efforts will ensure that the range of essential viewpoints are included.

### Initiative #3: Building a more diverse workforce

Improving cultural safety among physicians enhances the quality of healthcare for underrepresented populations [41]. A diverse and representative healthcare workforce has been shown to improve patients’ access to care, their perceptions of the care they receive, and their health outcomes, especially for patients from underrepresented groups in medicine [41–43]. It is important to have a healthcare workforce that represents the tapestry of communities to render the best possible care to diverse patient populations [43].

Black, Hispanic, and Native American people are underrepresented in healthcare professions [44]. Most health professions saw increases in applicants, matriculants, and degrees conferred from 2003 to 2019 from those in underrepresented groups in medicine; however, all programs were still below the age-adjusted US Census data [45]. The increased racial, ethnic, and gender diversity in the programs illustrates progress.

Tremendous progress has been made over 40 years since the 1985 Report of the Health and Human Services (HHS) Secretary’s Task Force on Black and Minority Health [46, 47]. It established the Office of Minority Health at the National Institutes of Health (NIH), among other accomplishments. The accumulated evidence of broader societal gains from these efforts are now well-established within the US healthcare system and they have provided the basis for current efforts to create a workforce that more closely resembles the patients being cared for.

Targeted outreach and scholarships to promote choosing health professions are important for populations underrepresented in medicine, especially persons of color. Urologists working with Boston Scientific discussed various pathways for improving the representativeness of clinicians after seeing how many young people who watched the COVID-19 pandemic unfold in their communities decided to pursue healthcare as a profession.

Based on the discussions with urologists and research regarding the critical impact of exposure to healthcare professions, Boston Scientific established scholarships for schools to facilitate greater access and opportunities for people of color looking to enter the healthcare workforce. The company sponsored the American Urological Association (AUA)’s Boston Scientific Medical Innovation Fellow in 2022–2024. This fellowship provides stipends to medical students from underrepresented groups in urology who are passionate about translating urology research into real-world solutions and matched them with world-renowned urologists. For 2024–2025, the company sponsored a post-residency fellowship through the Endourological Society for a female practitioner in endourology who met the underrepresented in medicine (URiM) definition per the Association of American Medical Colleges (AAMC). Also, beginning as a sole sponsor in 2023, the company supported the AUA FUTURE program, a longitudinal AUA mentoring program for mentoring those interested in pursuing urology, with a focus on underrepresented in medicine. At the inaugural program at the 2023 Annual Scientific Meeting of the AUA, there were approximately 40 medical student participants; in the second year in 2024, over 70 medical and premedical students participated and this has continued to flourish with newly established mentoring relationships spanning year-round outside of the scientific meeting. Similarly, the company has supported the Michigan Youth Summit from 2023–2024 to inspire middle school students from underrepresented communities to enter the field of medicine and possibly specialize in urology by supporting the summit with opportunities to interact with practitioners through hands-on demonstrations [48].

Boston Scientific’s Women in Stone initiative began in 2016 with a recognition of the need to increase the number of women focused on kidney stone and other urological stone diseases and the uniqueness of the barriers they face. The number has doubled from 30 to approximately 60; however, there is still work to be done to inspire women to specialize in the treatment of kidney stones. Meetings have occurred in-person and remotely at various national and global conferences and tradeshows, enabling connection and collaboration on topics such as the importance of ergonomics in kidney stone and other urological stone devices (lithotripters, lasers, ureteroscopes, etc.). Educational forums for women in urology have been sponsored at annual meetings of the Endourological Society and World Congress of Endourology.

In 2018, recognition that there were different challenges and barriers faced by women in prosthetic urology prompted Boston Scientific to gather and support an insight panel of 12 female physicians who were performing prosthetic urological surgeries. Women in Prosthetic Urology (WIPU) has grown to approximately 170 members who participate in insight panels and attend local and national gatherings to achieve the following goals: (1) elevate the voices of women in prosthetic urology; (2) promote cross-collaboration on topics such as patient talk tracks, operating room (OR) tips and tricks; and (3) create a network of female physicians for mentorship. In 2023, Boston Scientific supported the Society of Women in Urology, with new programs at AUA Sectional meetings, engaging over 200 physicians. The company is committed to supporting efforts that inspire underrepresented populations in medicine to engage in health professional society leadership through partnering with the Urology for Society Responsibility group and executing Pan Urology Society of Women in Urology (SWIU)/AUA Section programs and subspecialist programs.

### Initiative #4: Generating the evidence base for equitable care

Consideration of social determinants of health in the conduct of clinical and real-world research must occur [49]. There is a paucity of clinical trial data and real-world evidence (RWE) regarding disparities in diseases, diagnostics, treatments, and outcomes, particularly in the US. Pragmatic RWE assesses disease burden and how interventions work in real life, where patients are not carefully selected and may be sicker than patients in clinical trials or have trouble adhering to treatment plans. RWE helps consumers, clinicians, purchasers, and policymakers make more informed decisions that will improve healthcare at the individual and population levels. Boston Scientific is making special efforts to conduct RWE research studies in diverse populations of patients and to conduct research to gain a better understanding of the impact of gender, racial, ethnic, and socioeconomic disparities on disease burden, treatment patterns, access to care, healthcare resource utilization, and patient outcomes [50].

Evidence must be generated from randomized controlled trials (RCTs), real-world prospective clinical studies, retrospective chart and medical record reviews, retrospective database analyses, and health economic studies that are derived from underrepresented patient populations with the greatest needs. Diversity planning in clinical trials must come as the first step in achieving this goal, and specific efforts for representativeness are an important priority at the company. In the US, people of color make up 42% of the population [51]; however, they are consistently underrepresented in clinical trials. For example, although Black men represent approximately 13.4% of the US male population, they represent 6.7% of patients enrolled in therapeutic prostate cancer clinical trials [52]. The lack of diversity makes it challenging to evaluate the effects of treatments among different populations and even harder to mitigate the equity gaps accordingly [53]. Information regarding demographic representation is also frequently missing from studies [54].

The US FDA has issued the *Diversity Action Plans to Improve Enrollment of Participants from Underrepresented Populations in Clinical Studies* [55]. In a related view, Boston Scientific has actively established an initiative focused on advancing clinical trials for enhancing diversity, which implements innovative strategies to increase the enrollment of women and underrepresented patients [performance]. Leveraging this initiative, the company received FDA approval to expand its ELEGANCE study, from 1500 to 2500 participants. This study examining the real-world use of drug-coated devices for patients with PAD, was originally designed to ensure enrollment representation of at least 40% women and 40% underrepresented people of color, a benchmark it previously surpassed [56]. Similarly, Boston Scientific also aligns with the FDA diversity plan in ongoing urology trials [57].

With traditional RCTs taking time to develop, an alternative evidence-based approach to promote equitable care could be derived from non-traditional real-world data, such as administrative claims data analysis. This method could highlight inequities and shed light on critical clinical areas. Boston Scientific implemented proactive initiatives to utilize existing administrative claims databases to address diversity issues in research within a relatively short timeline. Some RWE efforts by expert urologists and the company include analyses of administrative databases that include socioeconomic variables (e.g., Medicare, Marketscan Medicaid, Department of Veterans Affairs [DVA] analyses, etc.). These real-world population-level studies have helped identify areas where disparities exist through analyzing healthcare utilization across various groups and demographics. Recently, the research found disparities in access to treatment for heart failure (HF) [58], coronary artery disease (CAD) [59], venous thromboembolism (VTE) [60], benign prostatic hyperplasia (BPH) [61], urinary incontinence (UI), and erectile dysfunction (ED) [62, 63]. Results from this research may help policymakers and healthcare providers in selecting healthcare interventions that provide the most equitable distribution of limited resources.

Evidence exists that incorporating social determinants of health into treatment have improved outcomes and delivered notable returns on investment [64]. Evidence also suggests that the costs of inaction, as we witnessed during the COVID-19 pandemic, are higher because we had not adequately addressed health inequities [65]. The Commonwealth Fund and the SCAN Foundation have coordinated resources to help support return-on-investment (ROI) estimates with freely available tools [66]. These ROI estimations need to be broadly defined to include other returns to the community beyond healthcare [66]. The difficulty of implementing ROI analyses for health equity investments is recognized and has been summarized in recent reports including: the importance of evaluating direct and indirect costs and returns, opportunity costs now and in the future, and the gains from a healthier expanded workforce [64, 65, 67, 68]. The evolution of the Triple Aim to the Quintuple Aim also provides a framework for how ROI can be considered, including value-based reimbursement [26, 69].

## Conclusions

No single individual, organization, community, or sector has sole responsibility for the health and well-being of an entire population [25]. Rather, multiple organizations, public and private, can positively influence social determinants of health by collaborating. Public–private collaborations are essential to addressing systemic shortfalls because, as described by the CDC, they “do more with less, build on the capabilities of others, leverage collective action, improve performance, and realize cost savings” [70]. Using diversity planning, health equity, and CSR strategies, businesses are strongly positioned to contribute to the improvement of the health and well-being of society at large, furthering the movement toward health equity [1]. Healthcare stakeholders such as urologists and healthcare companies working together can lead in efforts to help close the equity gap through actionable initiatives for delivering change. The ongoing four initiatives of healthcare stakeholders, in partnership with a global healthcare technology company, illustrate specific efforts that can help close the health equity gap and shape the future of healthcare to benefit everyone. Other industries are also well positioned to mirror the model of the four initiatives highlighted in this paper to positively impact health equity as well.

## Data and materials availability

This viewpoint article was based on iterative literature reviews, clinical expert experiences, and input from company employees and external health professionals collaborating on efforts to advance health equity.

## Data Availability

No datasets were generated or analyzed during the current study.

## Abbreviations

AAMC: Association of American Medical Colleges
AI: Artificial intelligence
AUA: American Urological Association
BDP: Breakthrough Devices Program
BPH: Benign prostatic hyperplasia
CAD: Coronary artery disease
CDC: Centers for Disease Control and Prevention
ConDUC: Consortium on Disparities of Urologic Conditions
CSR: Corporate social responsibility
DVA: Department of Veterans Affairs
ED: Erectile dysfunction
EMA: European Medicines Agency
FDA: Food and Drug Administration
LGBTQIA +: Lesbian, Gay, Bisexual, Transgender, Queer, Intersex, and Asexual + all other identities
OR: Operating room
PAD: Peripheral artery disease
PRO: Patient-reported outcomes
RCT: Randomized controlled trial
RFJUS: R. Frank Jones Urological Society
RWE: Real-world evidence
SWIU: Pan Urology Society of Women in Urology
UCSD: University of California, San Diego
UI: Urinary incontinence
US: United States
VTE: Venous thromboembolism
WIPU: Women in Prosthetic Urology
